# A case of synchronous triple autoimmune disorders secondary to thymoma: Pure red cell aplasia, Good's syndrome, and thymoma-associated multi-organ autoimmunity

**DOI:** 10.1016/j.rmcr.2022.101619

**Published:** 2022-02-23

**Authors:** Yuta Nakagawa, Kinnosuke Matsumoto, Makoto Yamamoto, Haruhiko Hirata, Takayuki Shiroyama, Kotaro Miyake, Yuji Yamamoto, Tomoki Kuge, Midori Yoneda, Yujiro Naito, Yasuhiko Suga, Kiyoharu Fukushima, Shohei Koyama, Kota Iwahori, Izumi Nagatomo, Yoshito Takeda, Atsushi Kumanogoh

**Affiliations:** a Department of Respiratory Medicine and Clinical Immunology, Graduate School of Medicine, Osaka University, 2-2 Yamadaoka, Suita City, Osaka, 565-0871, Japan; b Department of Immunopathology, WPI, Immunology Frontier Research Center (iFReC), Osaka University, 3-3-1 Yamadaoka, Suita City, Osaka, 565-0871, Japan; c Integrated Frontier Research for Medical Science Division, Institute for Open and Transdisciplinary Research Initiatives(OTRI), Osaka University, 2-1 Yamadaoka, Suita City, Osaka, 565-0871, Japan; d Center for Infectious Disease for Education and Research (CiDER), Osaka University, 2-8 Yamadaoka, Suita City, Osaka, 565-0871, Japan

**Keywords:** Thymoma, Pure red cell aplasia, Good's syndrome, Thymoma-associated multi-organ autoimmunity

## Abstract

Pure red cell aplasia (PRCA), Good's syndrome (GS), and thymoma-associated multiorgan autoimmunity (TAMA) are associated with thymoma. Herein, we describe the case of a 56-year-old woman with PRCA, GS, and TAMA simultaneously. She was treated with cyclosporine, immunoglobulin supplementation, and prednisolone; however, she died of uncontrolled sepsis due to extreme immunosuppression. The combination of these three diseases is likely to lead to fatal infections, and to avoid such infections, it may be necessary to reduce or discontinue immunosuppressants and steroids as soon as possible if the diseases are controlled, as well as regular immunoglobulin supplementation.

## 1 Introduction

Thymoma is associated with various autoimmune diseases, such as myasthenia gravis and pure red cell aplasia (PRCA). In addition, paraneoplastic disorders such as Good's syndrome (GS) and thymoma-associated multi-organ autoimmunity (TAMA) are rare [1,2]. PRCA is observed in 2–5% of thymomas, for which thymectomy and immunosuppressants, particularly cyclosporine, are effective [3].

GS is an immunodeficiency syndrome characterized by thymoma and hypogammaglobulinemia. There are a few reports where GS was improved by thymectomy and immunosuppressants; however, most cases are resistant to the above treatments. Therefore, antibiotics and immunoglobulins are generally administered only when infected [[4], [5], [6]]. It is unknown how much immunoglobulin should be administered to treat GS and at what time intervals.

TAMA is clinicopathologically similar to graft-versus-host disease (GVHD) and often presents with skin, liver, and gastrointestinal symptoms [2]. Treatment for TAMA is commonly a high-dose corticosteroid; however, lesions relapse in most cases as the steroid dose is decreased [7]. Therefore, steroids are often administered for long periods.

Although there are multiple case reports of PRCA, GS, and TAMA, there are no reports of a combination of the three at the same time. Here, we present the clinical course of three complications followed by thymoma.

## 2 Case presentation

**Fig. 1:**
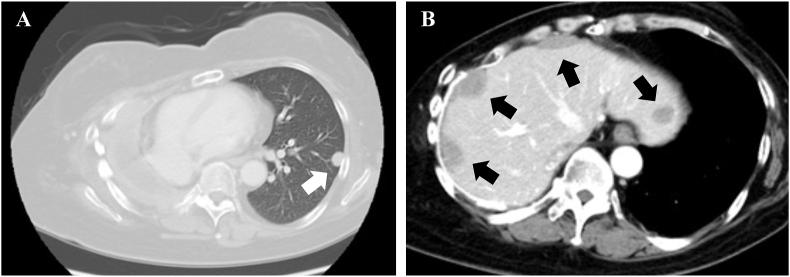
Chest computed tomography (CT) revealed multiple nodular lesions (A) in the left lung (white arrows) and (B) around the liver (black arrows) 8 years after complete resection of the thymoma with the right lung.

A 56-year-old woman with thymoma was admitted to our hospital with a XX-day history of dyspnea (day 1). Nine years earlier, she was diagnosed with a type B2 thymoma (modified Masaoka stage IVa) based on total thymectomy and pneumonectomy of the right lung, and received treatment with adriamycin, cisplatin, vincristine, and cyclophosphamide (ADOC). One year earlier, she was diagnosed with recurrence because of the appearance of new lesions in the left lung and peritoneum around the liver (Fig. 1). Therefore, the ADOC was rechallenged, leading to a partial response according to the Response Evaluation Criteria in Solid Tumors version 1.1 [8]. Nine months after the rechallenge with ADOC, the disease did not progress.

**Fig. 2:**
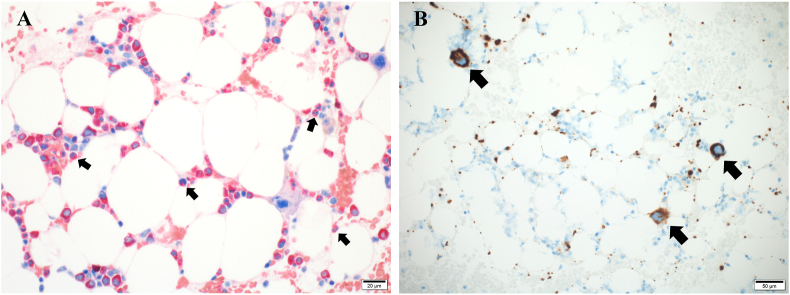
Bone marrow aspiration was performed on day 3. (A) NASD-CAE staining showed the lack of erythroid precursors (40 × 10) and the presence of granulocytes (black arrows) in a mature state. (B) CD42b staining showed the normal count of megakaryocytes (10 × 10).

The physical findings on admission were as follows: height 165.4 cm, weight 54.7 kg, body temperature, 36.8 °C; pulse rate 135/min, regular, blood pressure 91/73 mmHg. The conjunctiva was pale and the oral cavity lesions resembled candidiasis. The blood tests showed normocytic anemia with a hemoglobin level of 5.2 g/dl, a reticulocyte percentage of 0.1%, and the leukocyte and platelet counts were normal. The IgG level decreased to 405 mg/dl, and the beta-D-glucan and inflammation marker levels were elevated (Table 1). Examination of the bone marrow aspirate specimen revealed the absence of an erythroid component with normal granulopoiesis and megakaryopoiesis (Fig. 2).

**Table 1:** Laboratory findings on first admission.

Laboratory parameters	Value	Reference range
WBC	3,580	3,000–9,700 (/μl)
Platelet	14.9	12.4–30.5 ( × 10^4^/μl)
RBC	162	394-542 ( × 10^6^/μl)
Hemoglobin	5.2	13.1–17.6 (g/dl)
MCV	91.4	84.6–100.6 (fl)
MCH	32.1	28.0–34.6 (pg)
MCHC	35.1	31.6–36.0 (%)
Reticulocyte	0.1	2-26 (%)
Serum iron	166	50-170 (μg/ml)
Ferritin	572	5-152 (ng/ml)
Folic acid	5.1	3.9–26.8 (ng/ml)
Vitamin B12	1282	180-914 (pg/ml)
Coombs test	Negative	
Parvovirus B19	IgG (+)/IgM (−)	
IgG	405	820-1740 (mg/dl)
IgA	30	90-400 (mg/dl)
IgM	16	52-270 (mg/dl)
CRP	2.74	0.0–0.3 (mg/dl)
Beta-D-glucan	25.9	<20 (pg/ml)

WBC, white blood cells; RBC, red blood cells; MCV, mean corpuscular volume; MCH, mean corpuscular hemoglobin; MCHC, mean corpuscular hemoglobin concentration; IgG, immunoglobulin G; IgA, immunoglobulin A; IgM, immunoglobulin M; CRP, C-reactive protein.

**Fig. 3:**
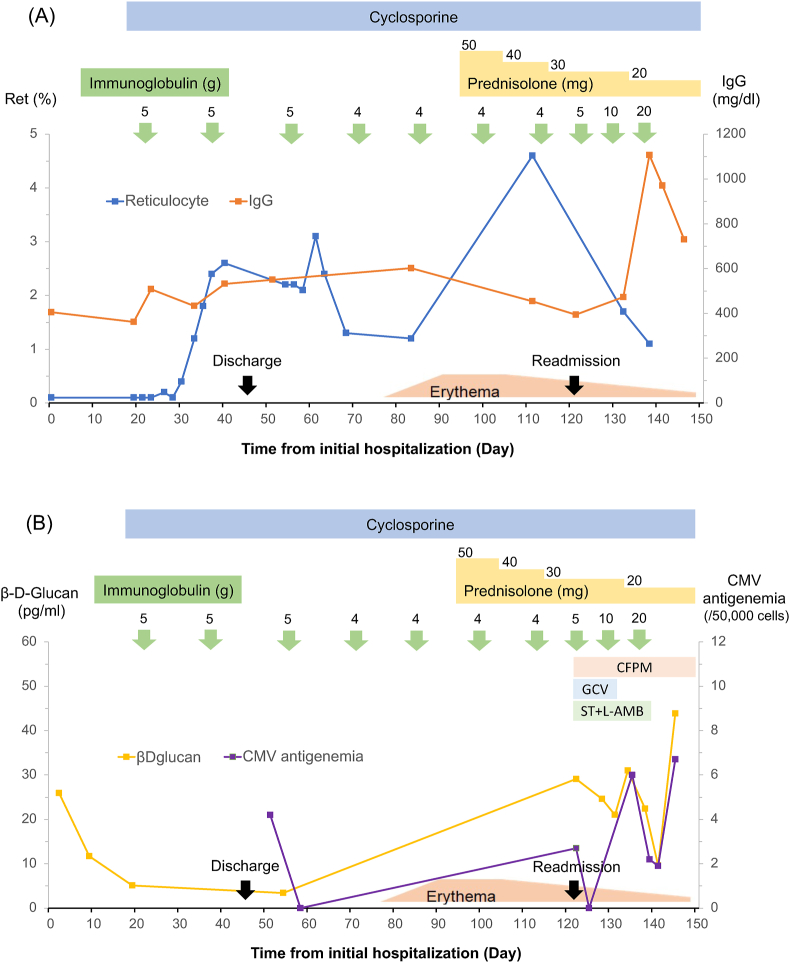
The clinical course. The patient was admitted with the chief complaint of suddenly dyspnea (day 1). She was diagnosed with PRCA and GS and started on cyclosporine and immunoglobulin on day 18. PRCA improved, and immunoglobulins were regularly replenished. Erythema appeared on day 77 and prednisolone 50 mg/day was started from day 95. The patient was urgently re-hospitalized due to severe pneumonia on day 122. She died of uncontrollable sepsis on day 152. (A) The level of reticulocyte gradually increased after cyclosporine started, and the level of IgG was kept around 500 mg/dl with immunoglobulin supplementations. (B) The level of beta-D-glucan and CMV antigenemia gradually increased after prednisolone started. CMV, cytomegalovirus; CFPM, cefepime; GCV, ganciclovir; ST, sulfamethoxazole-trimethoprim; L-AMB, liposomal amphotericin B.

Considering the clinical findings and thymoma, she was diagnosed with complications of PRCA and GS, and started cyclosporine (200 mg/day) as an immunosuppressant and received intravenous immunoglobulin (IVIG) (5 g) on day 18. PRCA gradually improved, whereas immunoglobulin replacement was regularly required for GS (Fig. 3). She was discharged due to improvement in reticulocyte and hemoglobin levels. IgG levels could be maintained at approximately 500 mg/dl due to the regular administration of immunoglobulins (4–5 g) approximately every 2 weeks. Beta-D-glucan levels decreased to normal without antifungal agents.

**Fig. 4:**
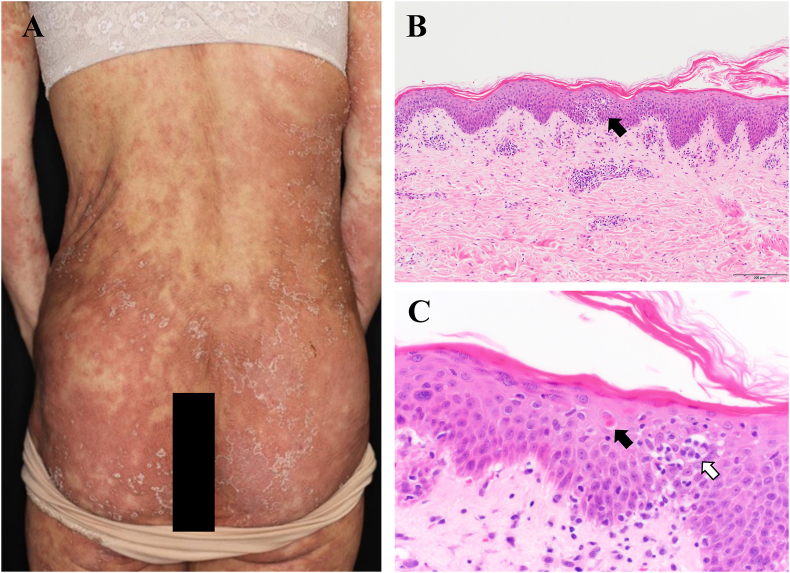
(A) Extensive erythematous with scaly plaque appeared over face, trunk, and extremities on day 86. Skin biopsy was performed on day 87. (B) Hematoxylin and eosin staining showed hyperkeratosis and cell infiltration in the upper dermis (black arrow) (10 × 10) and (C) necrotic keratinocytes (black arrow) and inflammatory cell consisting primarily of lymphocytes (white arrow) (40 × 10).

Extensive erythema with scaly plaques appeared over the face, trunk, and extremities on day 77, and then a skin biopsy was performed (Fig. 4). She was diagnosed with TAMA on the basis of physical findings, histopathology, and the presence of thymoma. Prednisolone (50 mg/day) was started in addition to the cyclosporine on day 95, which was tapered to 30 mg/day due to the skin improvement.

**Fig. 5:**
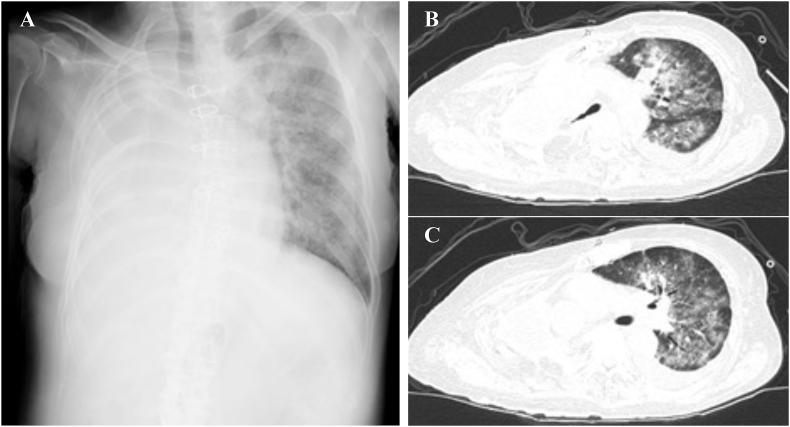
Chest X-ray and CT on urgent re-admission with complaints of high fever and severe dyspnea on day 122. (A) Chest X-ray showed the extensive ground-glass opacities in the left lung field. (B, C) Chest CT showed the diffuse ground-glass opacities in the left lung field and left pleural effusion.

On day 122, she presented with complaints of high fever and dyspnea and was intubated because of severe hypoxia. Chest computed tomography (CT) showed extensive ground-glass shadows in the left lung and left pleural effusion, and a blood test revealed increased cytomegalovirus antigenemia and beta-D-glucan (Fig. 5). *Pseudomonas aeruginosa* was detected in both sputum and blood culture tests. Combination treatment with cefepime, ganciclovir, sulfamethoxazole-trimethoprim, and liposomal amphotericin B was initiated immediately. Moreover, immunoglobulins were replenished on day 128 (IVIG 5 g) and day 130 (IVIG 10 g) because IgG levels hardly increased over 500 mg/dl. Although the IgG level was finally increased by administering IVIG at 20 g on day 138, it decreased to approximately half 10 days later. She then experienced repeated improvements and exacerbations of infectious diseases. Prednisolone was eventually tapered to 20 mg/day due to the stability of skin lesions; however, she suffered from uncontrollable sepsis and died on day 152.

## 3 Discussion

Herein, we present the clinical course of a patient who had severe infections due to multiple immunosuppressants, including cyclosporine for PRCA, immunodeficiency by GS, and steroids for TAMA. To the best of our knowledge, this is the first report of thymoma with PRCA, GS, and TAMA simultaneously.

Many authors have reported thymoma cases with PRCA, GS, or TAMA. One patient with GS received cyclosporine 100–200 mg/day and prednisolone 30 mg/day for acquired PRCA, resulting in severe infection and death [9]. Another patient with PRCA and myasthenia gravis developed TAMA and received cyclosporine 175 mg twice daily and prednisolone (40 mg/day). Although his skin improved, he died of multiple organ failure secondary to pneumonia [10]. From these reports, even when two of PRCA, GS, and TAMA are combined, a fatal infection may develop. We reviewed these diseases and suggested measures for recovery from such fatal infections.

Lesire et al. reported a systematic review of the management of thymoma-associated pure red cell aplasia [3,11]. In the literature, one of the most effective treatments is cyclosporine alone, with a remission rate of 83.9%; however, the optimal duration of cyclosporine administration remains unclear. Cyclosporine dose reduction or discontinuation may be associated with recurrence, and alternative treatments are quite limited in such cases [12,13]. Cyclosporine dose should be cautiously decreased or continued even after anemia is completely improved. In contrast, infection secondary to immunosuppression is the leading cause of death in PRCA [3]. In our case, cyclosporine was not interrupted because of concerns about a relapse of severe anemia like on day 1. However, such severe infections can lead to fatal conditions, and the interruption of immunosuppressants may need to be considered.

Second, immunoglobulin supplementation is effective in preventing infections in patients with common immunodeficiency disorders. Such patients have various trough IgG levels (500–1700 mg/dl) that prevent breakthrough bacterial infections, and immunoglobulin doses to prevent breakthrough infections range from 0.2 to 1.2 g/kg/month [14]. However, the amount of immunoglobulin that should be replenished in patients with GS is unknown. An IgG level of 500 mg/dl is said to be a guide to replenish immunoglobulin for hypogammaglobulinemia [15]. We also aimed to maintain IgG levels over 500 mg/dl and needed IVIG 0.08–0.1 g/kg every 2–3 weeks. Even though such an immunoglobulin dose resulted in severe pneumonia, a higher dose and more frequent supplementation may have been necessary. The guidelines of the Committee for Medicinal Products for Human Use (CHMP) recommend that the immunoglobulin dose is 0.2–0.4 g/kg every 3–4 weeks for secondary immunodeficiencies (defined as serum IgG level <400 mg/dl) [16]. Our patient had multiple forms of immunosuppression caused by cyclosporine use, steroid use, and GS; therefore, we suggest that in such cases, the immunoglobulin dose recommended by CHMP may need to be administered.

Finally, some studies have demonstrated the efficacy of steroids for TAMA; however, lesions are likely to recur when the steroid dose is reduced [17]. Moreover, patients with TAMA often die of pneumonia and septic shock. This is a result of the prolonged use of steroids because of the repeat of remission and relapse. Considering the possibility of recurrence, it was difficult to quickly reduce the steroid dose. In our case, high-dose prednisolone was unavoidable even when the patient was hospitalized for severe pneumonia. Alternative treatments for steroids include thymectomy, chemotherapy, and narrowband ultraviolet B phototherapy [18,19]. In our case, narrow-band ultraviolet B phototherapy could not be administered because of cyclosporine use, and chemotherapy could not be performed due to the absence of progression of thymoma. In patients with TAMA, steroid use may be a poor prognostic factor; therefore, it may be desirable to control thymoma as directly as possible [19].

## 4 Conclusion

In conclusion, we experienced a case of thymoma with PRCA, GS, and TAMA simultaneously. The combination of these three diseases is likely to lead to extreme immunodeficiency and fatal infections. To avoid such severe infections, the use of immunosuppressants, immunoglobulins, and steroids should be reviewed. It goes without saying that regular uses of immunoglobulins are indispensable, and immunosuppressants and steroids may be desirable to reduce or discontinue as soon as possible if the diseases are controlled.

## Author contributions

Conceptualization, investigation, recourses, and data curation: Yuta Nakagawa, Kinnosuke Matsumoto.

Writing-original draft: Yuta Nakagawa.

Writing-Review & Editing: Kinnosuke Matsumoto, Makoto Yamamoto, Haruhiko Hirata, Takayuki Shiroyama, Koutaro Miyake, Yuji Yamamoto, Tomoki Kuge, Midori Yoneda, Yujiro Naito, Yasuhiko Suga, Kiyoharu Fukushima, Shohei Koyama, Kota Iwahori, Izumi Nagatomo, Yoshito Takeda, Atsushi Kumanogoh.

Supervision and project administration: Haruhiko Hirata, Takayuki Shiroyama, Atsushi Kumanogoh.

## Declaration of competing interest

All authors declare no conflict of interest.
